# Budget Impact Analysis of Switching to Digital Mammography in a Population-Based Breast Cancer Screening Program: A Discrete Event Simulation Model

**DOI:** 10.1371/journal.pone.0097459

**Published:** 2014-05-15

**Authors:** Mercè Comas, Arantzazu Arrospide, Javier Mar, Maria Sala, Ester Vilaprinyó, Cristina Hernández, Francesc Cots, Juan Martínez, Xavier Castells

**Affiliations:** 1 Epidemiology and Evaluation Department, Hospital del Mar, Barcelona; IMIM (Hospital del Mar Medical Research Institute), Barcelona, Spain; 2 Red de Investigación en Servicios de Salud en Enfermedades Crónicas (REDISSEC), Valencia, Spain; 3 Gipuzkoa Oeste Research Unit, Hospital Alto Deba, Arrasate, Spain; 4 Basic Medical Sciences Department, Biomedical Research Institute of Lleida (IRBLLEIDA)-University of Lleida, Lleida, Spain; 5 Management Control Department, Hospital del Mar, Barcelona; IMIM (Hospital del Mar Medical Research Institute), Barcelona, Spain; 6 Radiology Department, Hospital del Mar, Barcelona, Spain; Geisel School of Medicine at Dartmouth College, United States of America

## Abstract

**Objective:**

To assess the budgetary impact of switching from screen-film mammography to full-field digital mammography in a population-based breast cancer screening program.

**Methods:**

A discrete-event simulation model was built to reproduce the breast cancer screening process (biennial mammographic screening of women aged 50 to 69 years) combined with the natural history of breast cancer. The simulation started with 100,000 women and, during a 20-year simulation horizon, new women were dynamically entered according to the aging of the Spanish population. Data on screening were obtained from Spanish breast cancer screening programs. Data on the natural history of breast cancer were based on US data adapted to our population. A budget impact analysis comparing digital with screen-film screening mammography was performed in a sample of 2,000 simulation runs. A sensitivity analysis was performed for crucial screening-related parameters. Distinct scenarios for recall and detection rates were compared.

**Results:**

Statistically significant savings were found for overall costs, treatment costs and the costs of additional tests in the long term. The overall cost saving was 1,115,857€ (95%CI from 932,147 to 1,299,567) in the 10^th^ year and 2,866,124€ (95%CI from 2,492,610 to 3,239,638) in the 20^th^ year, representing 4.5% and 8.1% of the overall cost associated with screen-film mammography. The sensitivity analysis showed net savings in the long term.

**Conclusions:**

Switching to digital mammography in a population-based breast cancer screening program saves long-term budget expense, in addition to providing technical advantages. Our results were consistent across distinct scenarios representing the different results obtained in European breast cancer screening programs.

## Introduction

In Spain, all resident women aged 50–69 are actively invited to participate in the population-based breast cancer screening program by written letter every 2 years. A screening mammogram (a type of low-dose x-ray examination used to detect breast cancer) is offered, allowing women who begin screening at 50–51 years up to a maximum of 10 screening mammograms. Breast cancer screening in Spain adheres to the European Guidelines for Quality Assurance in Mammographic Screening [1] and its results meet the required standards [2], [3].

Several studies have shown that digital mammography is more expensive than screen-film mammography [4]–[7], requires a considerable initial financial outlay and that the cost reimbursement of the switch is marginal. Previous studies have also emphasized the benefits of the technical features of digital mammography: the image is visualized in a computer window instead of a hard copy, which precludes film processing, storage, copying and retrieval. These benefits revert at the logistic level within a screening program: mammograms are visualized, stored and retrieved more easily, and allow radiologists to manipulate the image (such as zooms or changes of brightness and contrast) without additional exposure of the woman to radiation and attendance to the hospital. Digital equipment was approved by the US Food and Drug Administration based on results showing similar efficacy to conventional mammography [8]. The European guidelines for breast cancer screening recognize that digital mammography is likely to become established due to its advantages [1]. However, the cost-effectiveness of this switch in population-based screening programs, which involve millions of women and millions of tests, is controversial and, to our knowledge, no budget impact analysis has been performed to date.

In Spain, screening units are increasingly switching to digital mammography as a consequence of digitalization of radiology departments. The US Preventive Services Task Force recommendations [9], however, were based on the results of screening with screen-film mammography and consider that there is insufficient evidence to recommend digital mammography. The existing evidence on the effect of digital mammography in population-based breast cancer screening programs indicators is inconclusive: although the latest studies have found a slightly higher detection rate (number of cancers detected per 1,000 examinations) with digital mammography [10], the main conclusion of review studies is that digital mammography is at least as good as screen-film [11]–[13]. Recent studies in Spain [14]–[16] have found a lower recall rate (percentage of women called back for further tests) for digital mammography and a similar cancer detection rate. In contrast, other studies have found significantly higher recall rates with digital mammography [17], [18]. Further recent findings indicate the number of invasive procedures is lower with digital mammography [16], [19] and that the tumoral characteristics detected [16], [20] and interval cancer (primary breast cancer arising after a negative screening episode and before the next invitation to screening or within 24 months for women who reached the upper age limit) rates [21] are similar.

Budget impact analysis aims to estimate the impact of introducing a new technology in the budgets for the coming years [22]–[25]. According to Mauskopf et al. [22], this type of analysis measures the impact of a new technology on the annual cost and annual health benefit, as well as other outcomes of interest, in the years after its introduction in a national health system or a private health plan. For the time being, Budget Impact Analysis has had a short run in the scientific literature since the format used generally consisted in simple models based on assumptions from the literature and often on expert opinion. In recent years several authors have proposed guidelines for its development with more stringent requirements and have provided the scientific status to Budget Impact Analysis [22]–[25]. This type of analysis is especially important when assessing population-based programs in which small variations may affect a substantial proportion of the population.

In light of current findings, the economic impact of switching to digital mammography needs to be assessed. This impact includes not only the costs of mammography itself, but also the costs derived from performing a population-based screening program involving millions of women and tests, i.e., the costs of additional diagnostic tests and those of cancer treatment. The objective of the present study was to estimate the long-term impact on costs and health outcomes of switching from screen-film mammography to full-field digital mammography in a population-based breast cancer screening program from the perspective of a National Health System.

## Methods and Materials

### Ethics Statement

This study was approved by the ethics committee of Hospital del Mar. The need for written informed consent was waived because all data was analyzed anonymously.

### Discrete-event simulation model

**Figure 1:**
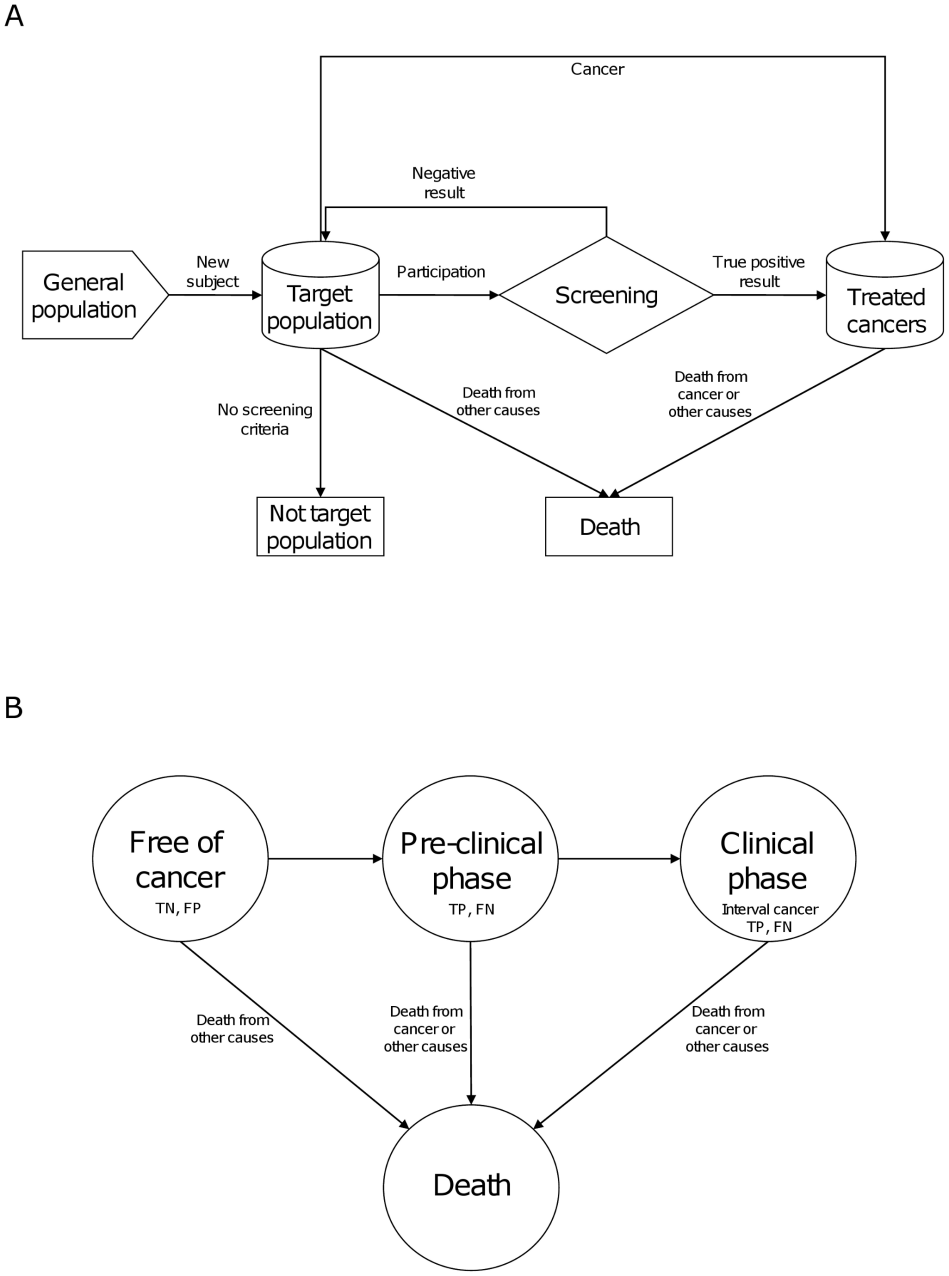
Flow chart of the conceptual model (screening [a] and natural history of cancer [b]). TN: true negative, FP: false positive, TP: true positive, FN: false negative.

Discrete-event simulation has been defined as “a flexible modeling method characterized by the ability to represent complex behavior within, and interactions between individuals, populations, and their environments” [26]. A discrete-event simulation model was built to reproduce the process of women entering a population-based breast cancer screening program. The events simulated were as follows: inclusion of a new woman in the target population, participation in the screening program, a positive or negative result of screening (including screening mammography and additional tests), nonscreening cancer detection, exit from the target population, and death. The conceptual model was based on the European guidelines [1] and is depicted in Figure 1.

Parallel to the events related to the screening program, each woman was assigned a natural history of breast cancer. These events consisted of the start of the pre-clinical stage (when the cancer appears but it is asymptomatic), the start of the clinical stage (when the cancer is symptomatic), and death (Fig. 1). The model allowed for the absence of progression but did not consider spontaneous cancer remission.

The time units were years. A simulated time horizon of 20 years (from 2010 to 2029) was chosen to encompass the life history of a woman entering a screening program (from 50 to 69 years) and to allow the budget impact to be analyzed in the long term. Individual women were simulated. Two identical groups of women with the same pattern of screening participation were simulated: one group by using the parameters estimated for screen-film mammography and the other by using parameters for full-field digital mammography. The times until an event of the natural history of breast cancer were also the same unless modified by cancer detection. All women underwent biennial screening from 50 to 69 years of age. Women aged 70 years or older were followed-up until 2029 only if they were diagnosed with cancer between the ages of 50 and 69 years or with an interval cancer in the last mammogram.

The simulation model was implemented by using Arena (Rockwell Software) version 13.9.

### Target population

The target population at the beginning of the simulation included 100,000 women aged 50-69 years undergoing biennial screening. Every 2 years, women aged 50–51 years old entered the target population, following the age structure of the Spanish population [27]. Women undergoing their last mammogram (aged 68–69) were excluded from the target population unless they were found to have breast cancer within the screening program or an interval cancer in the last mammogram (details of the estimation of the parameters and the simulation are provided in Appendix S1).

### Natural history model parameters

Breast cancer incidence [28] was used to estimate the time until pre-clinical onset [29], [30]. The duration of pre-clinical status was modeled according to age at pre-clinical onset [31], ranging from 2 in 40-year-olds and increasing to 4 years after that age of 50 years (see the appendix S1 for further details).

Age of death from any cause was modeled using the number of women and the number of deaths, by age, of the Spanish population in 2008 [27], [32].

We assumed that if a woman enters the clinical stage, the cancer is detected on the basis of symptoms. Currently available data did not allow modeling the natural history of breast cancer based on progression through cancer stages, occurrence of symptoms or recurrences. Thus, the cancer stage was assigned according to the distributions described in the appendix S1 for screening-detected and clinically-detected cancers. Survival according to stage and age at detection also depended on the birth cohort [33] (see the appendix S1 for further details).

### Screening events

The participation profile for each woman was generated at the beginning of the simulation in order to ensure the same participation profile for digital and screen-film screening. Participation was treated as a probabilistic parameter (see appendix S1 for details) with values based on a probability of 78.7%for participation in initial screening and of 83.2% in successive screenings. When a woman was scheduled to participate in a screening round, a result was sampled using sensitivity values for women in the pre-clinical or clinical stage and specificity values for cancer-free women.

The proportion of false-negative results was obtained from a study on interval cancers [34]. False-negatives were defined as all interval cancers not classified as true interval cancers and included existing cancers not detected by mammography: false negatives, occult tumors, and minimal signs, which all together represent 57.5% of false negatives [34]. This percentage was applied to the overall number of interval cancers in the 20-year period and sensitivity was calculated. Sensitivity was assumed to be similar for both techniques [10]–[13] but was modeled as a probabilistic parameter and was sampled separately for each technique using the same statistical distribution with a mode of 86.66%. This allowed us to obtain scenarios with similar and different sensitivities favoring digital or screen-film mammography (see appendix S1).

Specificity was also treated as a probabilistic parameter and was calculated by stratifying by digital or screen-film mammography as well as by initial or successive screening, giving results of 88.8% for digital and 88.3% for screen-film mammography in the initial round and 95.8% for digital and 95.4% for screen-film screening mammography in successive rounds [15]. These results coincided with the mode of the distributions for specificity (see appendix S1) and also allowed us to obtain scenarios with similar and different specificities favoring one or other technique.

In breast cancer screening, if a screening result is positive, a combination of additional (confirmatory) tests is assigned to the woman. Tests include additional mammographic projections, ultrasound, fine-needle aspiration cytology (FNAC), core biopsy, and open surgical biopsy. By using the Breast Cancer Screening Program database, the relative frequencies of the existing combinations of tests were calculated, stratifying by digital or screen-film mammography as well as by initial or successive screening.

### Cancer detection

In our model cancer is detected inside and outside the screening program. The latter refers to nonparticipating women and interval cancers. The distribution of cancer stage at detection differs according to age and the screening round (initial or successive) as well as according to the detection setting. Clinical detection refers to cancers detected by clinical symptoms (interval cancers or cancers in nonparticipating women) and screening detection refers to those detected through a screening program (participating women). The stage distribution of screen-detected cancers differed according to digital or conventional mammography but that of clinical detected cancers was the same. In all cases, stage distribution was treated as a probabilistic parameter. See appendix S1 for more details.

### Unitary costs

The study was carried out from the perspective of a National Health System that includes in its portfolio both preventive programs and clinical treatments, as it is the case of Spain and the majority of European countries. That means that the same payer bears all the preventive and curative costs. The costs of both screen-film and digital mammography were obtained from the accounting system of a Spanish program [35] before and after the process of switching from screen-film to digital mammography. The cost per woman screened was 39.29€ with screen-film and 42.28€ with digital mammography. These figures included all the program costs: human resources, equipment and structure, and corresponded to year 2009 [35]. Amortization times for equipments (radiological and Picture Archiving and Communication System - PACS) were set at 10 years following accounting criteria.

The cost of diagnostic and screening mammograms was assumed to be the same, according to expert opinion. The reason is that, although a screening mammogram needs to be read by two radiologists and needs a higher number of projections, it takes an average of 3 minutes to read, while a diagnostic mammogram needs fewer projections and is read by one radiologist but reading takes longer.

The costs of breast ultrasound, FNAC, core needle biopsy and open surgical biopsy were obtained from the cost accounting systems of several Spanish hospitals[36]. Women identified as having had these procedures in the Breast Cancer Screening Program were analyzed. The cost of a breast ultrasound was 16.69€. The cost of an FNAC included the cost of the procedure (80.22€), the cytological analysis of the sample (80.78€) and the consultation with the surgeon or gynecologist to inform the woman of the result (142.25€), amounting to 303.25€. The cost of a core needle biopsy included the procedure (113.54€), the histological analysis of the sample (14.37€) and the consultation with the surgeon or gynecologist to inform the woman of the result (142.25€), amounting to 270.16€. The cost of an open surgical biopsy was 1,388€ [36].

The cost of treatment was obtained from a study [37] that included incident breast cancer patients from 2000 to 2005. Patients were followed-up until December 2007 and the costs of treatment were included in addition to other variables such as cancer stage. Three different phases were considered for stages lower than stage IV: initial phase, follow-up and, if there was a recurrence, advanced phase. For stage IV, only the advanced phase was considered. For each phase, the mean duration and mean cost per month were calculated. For stages lower than IV, the probability of recurrence was also calculated. Within the model, each woman with a diagnosis of cancer was assigned a monthly cost, according to the detection stage and treatment phase. The costs and durations of the phases are shown in Table 1. Original costs were based on the year 2005 and a cumulative Consumer Price Index (CPI) of 7.9% was applied to adjust them to the year 2009.

**Table 1:** Costs of cancer treatment per month, cancer stage and treatment phase.

		Initial phase		Follow-up		Advanced phase		
		Duration (months)	Cost per month	Duration (months)	Cost per month	Probability of recurrence	Duration (months)	Cost per month
Stage								
	CIS	5.75	2,006.25	115.25	132.10	2.75		
	I	9.40	2,006.25	111.60	291.87	7.85		
	II	10.62	2,236.92	110.38	577.35	14.68		
	III	11.11	2,236.92	109.89	737.65	35.38		
	IV						lifetime	3,438.71

CIS: Carcinoma in situ.

Similar monthly costs of the initial phase were found between DCIS and stage I, and between stages II and III.

The duration of the follow-up phase included the time from surgery until a maximum of 10 years, i.e. follow-up was calculated as 10 years (120 months) minus the duration of the initial phase except the first month.

The duration of follow-up for women assigned to have a recurrence was sampled from a Uniform distribution from the end of the initial phase to the end of the follow-up phase.

The costs of metastatic cancer were similar, regardless of whether the tumour was an initial stage IV cancer or a recurrence. Its duration was for the lifetime.

### Probabilistic sensitivity analysis

Crucial parameters such as sensitivity, specificity, the stage distribution of detected cancers and participation were included as probabilistic parameters (see Appendix S1 for details on the distributions assigned). The results of the sensitivity analysis were stratified according to groups. The groups were created, based on the detection and recall rate: a group of simulation runs with better rates for digital mammography and another group with better rates for screen-film mammography were established. A better detection rate was defined as a rate at least 0.5‰ higher and a better recall rate was defined as a rate of at least 0.5% lower. Another group was defined by including those runs with a better detection rate and a higher proportion of noninvasive cancers detected through screening (at least 1% higher) with digital mammography.

### Simulation analysis of results

Results were analyzed as the mean of 2,000 replications of the model with independent streams of random numbers. This sample size was calculated to detect a significant difference in overall costs at the 0.05 level using preliminary results with 100 replications, and allowed the results to be stratified for sensitivity analysis. The time units were years and the simulation horizon was 20 years, from 2010 to 2029.

The following results were used to validate the model: the number of invited women through time (by initial and successive screenings), the participation rate, the mean age of invited women, the number of mammograms over time (by initial and successive screenings), the recall rate, cancer detection rate, false-positive rate, false-negative and interval cancer rate, the distribution of additional tests, the distribution of cancer stage (pre-clinical and clinical), and life expectancy. Validation results were shown to the research team using graphics through time and compared to published and unpublished data on Spanish programs. The distributions of the probabilistic parameters were also shown and its relationship with outcomes explained, as well as published data from other countries was presented to contextualize the need to include them as probabilistic parameters. The research team checked the validation results and the model was considered as valid, credible and useful for the purposes of the study.

### Budget Impact Analysis

Budget impact analysis requires the inclusion of the entire population involved in a system each year [38]. Thus, individual women entering and exiting the model were simulated throughout the simulation horizon. Each year, the impact on the budget was calculated according to the difference between the two alternatives (screen-film minus digital) in overall costs, screening costs, and the costs of additional tests and cancer treatment. The cost results for each technique and the confidence intervals for the cost differences were shown for years 2010, 2015, 2020, 2025 and 2029. Costs were not discounted, according to the published good practices for budget impact analysis [38].

## Results

The model started with a target population of 100,000 women aged 50–69 years. Over the 20 simulated years, 151,960 women were dynamically incorporated into the target population, making a total of 251,960 women invited to the program and resulting in more than 731,400 screening mammograms, of which 15.3% corresponding to initial screening (Table 2).

**Table 2:** Twenty-year cumulative results on health and validation outcomes, according to type of mammogram.

		Digital mammography		Screen-film mammography	
		N	%	N	%
**Overall data**					
Invited women		251,960		251,960	
Initial population		100,000		100,000	
New women entering target population		151,960		151,960	
Screening mammograms		731,510		731,506	
Initial screening		111,718	15.3%	111,718	15.3%
Successive screening		619,792	84.7%	619,788	84.7%
**Recall for further assessment**					
Recall rate		44,536	6.1%	47,931	6.6%
Further assessments					
Additional mammograms		19,085	2.6%	28,529	3.9%
Ultrasound		34,241	4.7%	37,809	5.2%
Fine-needle aspiration cytology		11,812	1.6%	20,729	2.8%
Core biopsy		2,725	0.4%	4,611	0.6%
Open surgical biopsy		302	0.04%	1,544	0.2%
False positive rate		39,833	5.4%	43,226	5.9%
Initial screening		12,812	11.5%	13,275	11.9%
Successive screening		27,021	4.4%	29,951	4.8%
**Cancer detection**					
Cancer detection rate		4,702	0.643%	4,704	0.643%
Carcinoma *in situ*		1,008	21.4%	841	17.9%
Invasive cancers		3,694	78.6%	3,864	82.1%
Stage I		1,944	41.3%	2,073	44.1%
Stage II		1,380	29.4%	1,230	26.1%
Stage III		350	7.4%	549	11.7%
Stage IV		20	0.4%	13	0.3%
Interval cancer rate		1,588	0.217%	1,587	0.217%
True interval cancers (% of all cancers)		1,271	27.0%	1,271	27.0%
False negatives (% of all cancers)		316	6.7%	316	6.7%
**Mortality**					
Deaths due to cancer		1,887	0.749%	1,942	0.771%
Carcinoma *in situ*		16	0.9%	14	0.7%
Stage I		444	23.6%	463	23.9%
Stage II		790	41.9%	791	40.7%
Stage III		424	22.5%	519	26.7%
Stage IV		159	8.5%	155	8.0%

The recall rate was 6.1% for digital and 6.6% for screen-film mammography. Fewer additional tests were required with digital mammography (Table 2). The false positive rate was 5.4% for digital mammography and 5.9% for screen-film mammography. The cancer detection rate (0.64%), the interval cancer rate (0.22%) and the mortality rate (about 0.76%) were similar between digital and screen-film mammography (Table 2).

**Figure 2:**
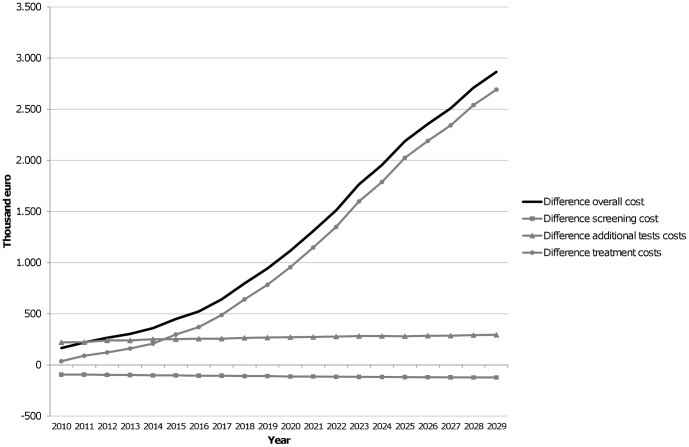
Budget impact analysis. Differences in cost between screen-film and digital mammography, by type of cost and year. Positive differences indicate cost savings with digital mammography.

Table 3 and Figure 2 show the budget impact of digital mammography in the short and the long term. The cost of the screening program with digital mammography was always higher but was offset by the savings due to a lower need for additional tests. Reductions in treatment costs were an additional saving. Figure 2 shows the yearly difference between the two screening modalities. Confidence intervals in Table 3 show significant savings in overall costs and the costs of additional tests and treatment in the short and long term. Moreover, the savings in treatment costs were clear and increased over time. The overall cost savings were 165,540€ (95%CI from 133,253 to 197,827) after 1 year, 1,115,857€ (95%CI from 932,147 to 1,299,567) at 10 years and 2,866,124€ (95%CI from 2,492,610 to 3,239,638) at 20 years. This saving represented reductions/reduced costs/savings of 3.0%, 4.5% and 8.1%, respectively, over the overall cost of screening with screen-film mammography.

**Table 3:** Budget impact analysis of digital mammography compared with screen-film mammography.

		Year			
	2010	2015	2020	2025	2029
Digital mammography					
Overall cost	5,429,104	15,546,355	23,922,992	29,002,023	32,420,266
Screening	1,318,641	1,437,720	1,591,541	1,671,728	1,722,550
Additional tests	254,246	290,016	316,109	328,451	334,096
Cancer treatment	3,856,217	13,818,618	22,015,343	27,001,844	30,363,620
					
Screen-film mammography					
Overall cost	5,594,644	15,996,017	25,038,849	31,190,348	35,286,390
Screening	1,225,388	1,336,029	1,478,975	1,553,505	1,600,717
Additional tests	475,898	542,930	588,531	609,703	629,704
Cancer treatment	3,893,358	14,117,058	22,971,343	29,027,141	33,055,968
Difference (Screen-film - Digital)					
Overall cost	165,540	449,662	1,115,857	2,188,325	2,866,124
95%CI	[133,253; 197,827]	[344,495; 554,830]	[932,147; 1,299,567]	[1,897,187; 2,479,463]	[2,492,610; 3,239,638]
Screening	−93,253	−101,692	−112,566	−118,224	−121,833
95%CI	[−93,517; −92,988]	[−101,966; −101,417]	[−112,873; −112,259]	[−118,544; −117,903]	[−122,167; −121,498]
Additional tests	221,652	252,914	272,423	281,252	295,608
95%CI	[217,116; 226,187]	[248,017; 257,812]	[267,096; 277,750]	[275,884; 286,620]	[289,974; 301,242]
Cancer treatment	37,141	298,440	956,000	2,025,297	2,692,348
95%CI	[5,298; 68,984]	[193,516; 403,364]	[772,553; 1,139,447]	[1,734,283; 2,316,311]	[2,319,115; 3,065,582]

CI: Confidence Interval.

**Figure 3:**
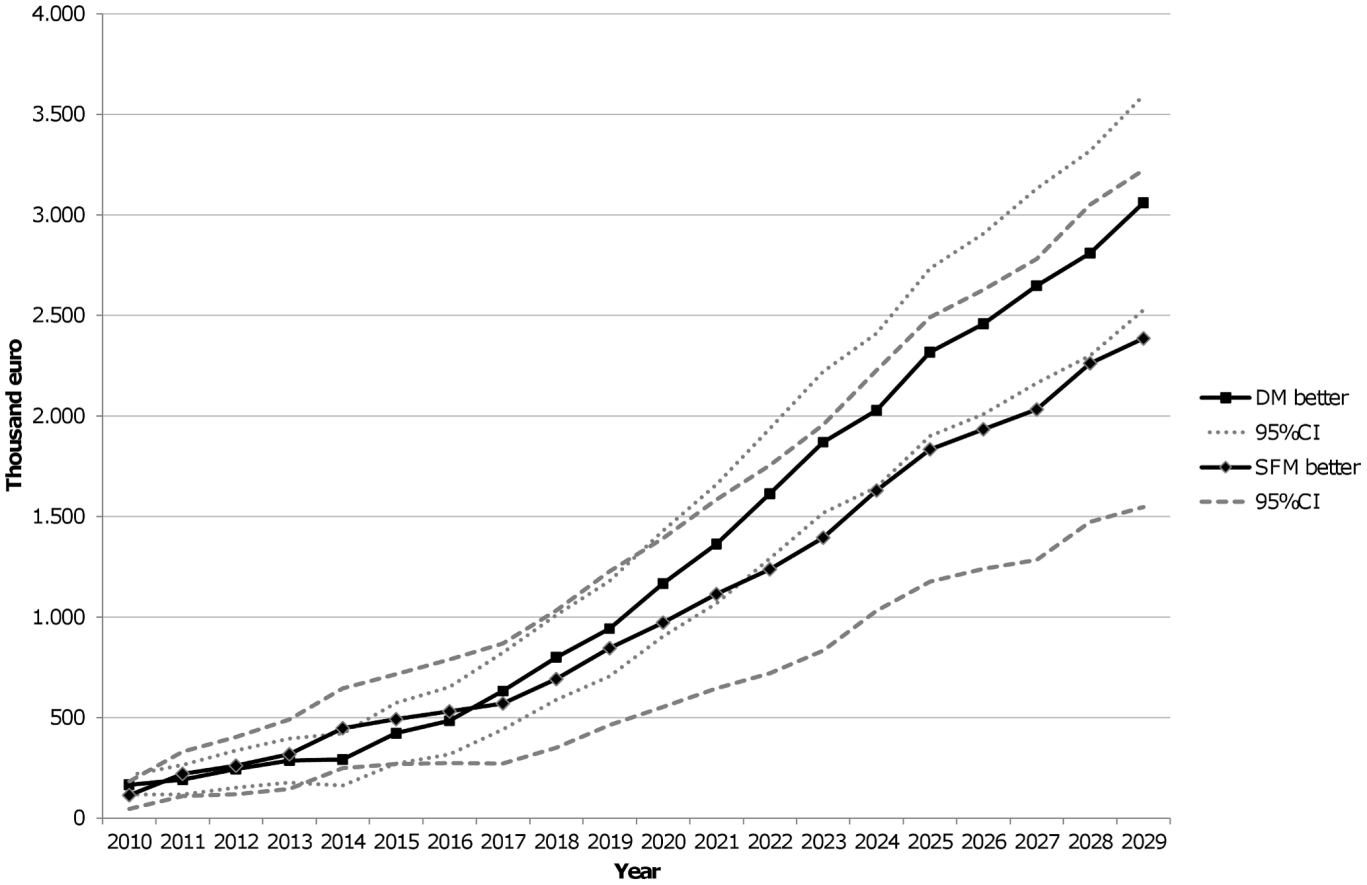
Sensitivity analysis results. DM better (n = 921 runs): Digital mammography higher detection rate and lower recall rate, or digital higher detection rate and similar recall rate, or digital lower recall rate and similar detection rate. SFM better (n = 469 runs): Screen-film mammography higher detection rate and lower recall rate, or SFM higher detection rate and similar recall rate, or SFM lower recall rate and similar detection rate. Intermediate scenario (n = 610 runs, not shown): Digital higher detection rate and SFM lower recall rate, or digital lower recall rate and SFM higher detection rate, or both similar detection and recall rates. CI: Confidence Interval.

Figure 3 shows the results of the sensitivity analysis for the groups of runs with better detection and recall rates for digital (n = 921) and for screen-film (n = 469) mammography. Both groups showed a significant net benefit of digital mammography.

The results of runs with a better detection rate and a higher proportion of noninvasive cancers with digital mammography showed higher costs of digital mammography in the short term, but significant savings in the long term (after the 10^th^ year, data not shown).

## Discussion

The main finding of the budget impact analysis was that a switch to digital mammography results in net savings if, in addition to screening costs, the costs of additional tests and treatment are taken into account, with similar screening results. The model was run for an initial population of 100,000 women aged 50–69 years, which, according to the current structure of the Spanish population, would correspond to an overall population of about 860,000 inhabitants.

Several studies [4]–[7] have reported that digital mammography is more expensive than screen-film mammography. However, the actual economic impact should take into account all the costs derived from a population-based screening program, that is, the costs of confirmatory tests and those of treating detected cancers.

To our knowledge, this is the first study to perform a budget impact analysis of digital mammography for breast cancer screening. Previous studies have consisted of cost comparisons [4], [7] and cost-effectiveness studies [5], [6] and have included the costs of screening only. Our results show that the savings due to fewer additional tests alone offset the higher cost of screening with digital mammography. In addition, the greatest savings corresponded to treatment costs.

Our model shows that, although the differences in recall rate between digital and screen-film mammography are small in magnitude, they result in substantial cost savings in the long term as the lower recall rate with digital mammography results in fewer adverse events and additional tests with a consequent reduction in the negative impact on women. The most frequent additional test with digital mammography was one ultrasound scan (about 30%), while for conventional mammography a combination of ultrasound and FNAC was used (about 22%). Because the differences in recall and detection rate between screen-film and digital mammography are controversial, sensitivity and specificity were included as probabilistic parameters, meaning that some simulations were run with better sensitivities and/or specificities for digital mammography, others were run with better sensitivities and/or specificities for screen-film mammography, other simulations were run combining opposite directions for each parameter and still others were run with similar parameters. The results of the sensitivity analysis performed with the results of detection and recall rates demonstrated that, for all subgroups, the switch to digital mammography produced significant net savings in the long term, indicating that our results are consistent and robust concerning differences in the detection and recall rates between digital and screen-film mammography. The sensitivity analysis stratified by excess detection of carcinoma *in situ* with digital mammography also showed net savings in the long term (data not shown), despite excess costs in the short term.

The model's parameters were mostly obtained from Spanish breast cancer screening programs that followed the standards of the European guidelines, thus conferring homogeneity to the model. However, the parameters related to the natural history of the disease, such as the sojourn time in the pre-clinical stage, although adapted to our population, were based on US data.

Treatment costs may have been underestimated because medication criteria corresponded to 2000–2005 and costs to 2005 and more expensive chemotherapy treatments have been introduced since then. In addition, treatment costs included those of women with cancers detected within the simulation horizon only. Although this consideration did not pose a limitation to our objective of comparing digital with screen-film mammography screening, it did lead to underestimation of treatment and overall costs, especially in the short term.

Given that our objective was to compare the population-based screening program using one technology versus the same program using the other, the costs and inefficiencies associated to the process itself of switching from screen-film to digital mammography have not been included [39]. Further research would be needed to estimate the economic impact of the process of change and how increased short-term costs may hamper implementation of digital mammography throughout all the Spanish screening units.

In conclusion, switching from screen-film to digital mammography within a population-based breast cancer screening program reduces expense in the long term in addition to providing technical advantages. The higher expense of digital screening is offset by the reduction in additional tests. These results were consistent across distinct scenarios representing the different results obtained in European breast cancer screening programs.

## Supporting Information

Appendix S1 **Supporting information to: Budget impact analysis of switching to digital mammography in a population-based breast cancer screening program.**
(DOCX)

Table S1 **Beta distributions for probabilistic sensitivity analysis.**
(DOCX)

Table S2 **Parameters of the Dirichlet distributions for cancer detection stages by age, detection setting, type of mammography and screening number.**
(DOCX)
