# Supplementary material for: Budget Impact Analysis of Switching to Digital Mammography in a Population-Based Breast Cancer Screening Program: A Discrete Event Simulation Model
Source: PLoS One. 2014 May 15;9(5):e97459. doi: 10.1371/journal.pone.0097459 (PMC4022526; doi:10.1371/journal.pone.0097459)
Supplement: Appendix S1 — Supporting information to: Budget impact analysis of switching to digital mammography in a population-based breast cancer screening program. (DOCX) [file pone.0097459.s001.docx]

**Appendix S1**

*Target population*

The target population at the beginning of the simulation included 100,000 women aged between 50 and 69 years. A warm-up period of 20 additional years was included in the model with the purpose of simulating natural history of cancer, cancer incidence and mortality in women from 30 to 49 years old during the warm-up period. These women would enter the program dynamically during the 20 years of results collection, following the age structure of the Spanish population [1] and with numbers proportional to the initial population of 100,000. Women who entered the clinical stage before the age of 50 were excluded from the screening path. The target population of women aged 50 years or older at the beginning of the simulation was that of round number 5 (years 2004-2005) of the Population-based Breast Cancer Screening of Hospital del Mar, Barcelona, Spain. This program includes about 36,000 women from two districts in the city of Barcelona. This sample was needed in order to have age and health status values for woman who had already started their screening history. Breast cancer screening in Spain adheres to the European Guidelines for Quality Assurance in Mammographic Screening [2] and its results meet the required standards [3,4].

A simple random sample of 10% of women aged 30 to 69 years was selected and formed the initial population. Women between 50-69 years were selected as the target population and were assigned a time to the next mammogram from a uniform distribution between 0 and 2. The model assumed that the program was able to screen all participant women within 2 years.

Every 2 years, women aged 50-51 years old entered the target population, following the age structure of the Spanish population [1]. A percentage of 11.51% was excluded due to reasons other than cancer, while 22.73% were assigned to have external screening (under private care or another screening program). For women initially forming the target population, exclusions were removed before selecting the random sample. External screening accounted for 19.23%. These percentages were calculated using the database of the fifth round of the Hospital del Mar screening program.

Women aged 50-51 years old who entered the model in a given round were uniformly distributed to have their first invitation/mammogram within 2 years. After the first mammogram, interval to the next mammogram was calculated by adding 2 years.

The probability that an invited woman would participate was calculated as the number of participants over the sum of participants and nonparticipants, excluding women who had external screening. The resulting values were 78.7% and 83.2% for initial and successive screening, respectively. These probabilities were also calculated using the database of the fifth round.

Women undergoing their last mammogram (aged 68-69 years) were excluded from the target population unless they were found to have breast cancer within the screening program or an interval cancer after the last mammogram.

*Natural history model parameters*

A breast cancer incidence model based on data from two population-based cancer registries of the Catalan provinces of Girona and Tarragona that cover 20% of the Catalan population [5] was used to estimate the time until pre-clinical onset [6]. The incidence of the pre-clinical stage was modeled as the time from the age of 30 years until onset of the pre-clinical stage. Quadratic models were the best-fitting option to model the incidence rate of pre-clinical stage by age and year of birth.

$$rate_{cohort}(t)=\beta_{0}+\beta_{1}t+\beta_{2}t^{2}$$

Where *t* is age and *cohort* takes the values 1955, 1960, 1965, 1970 and 1975.

The conversion to time to the event was done using basic formulae from survival theory. Then the cumulative probability distribution function, *F(t)*, was obtained.

$$F(t)=1-S(t)=1-\exp\left(-\int_{0}^{t} rate_{cohort}(u)du\right)=1-\exp\left(-\left[\beta_{0}t+\frac{1}{2}\beta_{1}t^{2}+\frac{1}{3}\beta_{2}t^{3}\right]\right)$$

This distribution function takes a long time to reach 1 because most women will not enter the pre-clinical stage during their lifetime. Therefore, for simplicity’s sake, and assuming that no women will reach the age of 150, *F(t)* was calculated from 30 years to 149 and the value of 1 was assigned for 150 years.

The pre-clinical sojourn time (time between pre-clinical onset and clinical onset) followed an exponential distribution with an age dependent mean [7]. The mean sojourn time in pre-clinical status values were:

$$\alpha(age)=\left\{\begin{matrix} 2 & & age\leq40 \\ -6+\frac{age}{5} & & 40<age<50 \\ 4 & & age\geq50 \end{matrix}\right.$$

Where *age* is the age at the pre-clinical onset. The sojourn time in the pre-clinical stage was based on US data.

We assumed that, if a woman enters the clinical stage, the cancer is detected on the basis of symptoms. If a woman entering the clinical stage had a screening mammogram scheduled within 1 month, the cancer was detected under the program. Then, the stage of detection of the cancer could be assigned according to the distributions depicted in Table S2. Given the stage and the age at detection, a Bernouilli distribution was sampled using the probability of breast cancer survival by age and cohort of birth [8]. If the result was survival, the age of death from any cause was not modified. If the result was nonsurvival, the age of death from cancer was sampled from the corresponding distribution [8]. Then, the age of death of the woman was substituted by the sampled age of death from cancer if the latter was lower.

Although adapted to the Catalan population, natural history parameters were based on US data. Breast cancer survival functions were adapted to the Catalan population by estimating the hazard rate of mortality between the USA and Catalonia with data for women diagnosed between 1990 and 2001 in Catalonia [8]. Because screening was implemented in Catalonia during this period, survival functions may have been affected by the lead time bias caused by earlier detection. However, this bias would have had the same effect on the results for both digital and screen-film mammography.

The age of death from any cause was sampled from a Gompertz distribution [9]. The parameters were estimated using the number of women and the number of deaths, by age, of the Spanish population in 2008. Considering time until death from causes other than breast cancer to be similar to time until death from all causes would introduce a negligible bias, which would have the same effect on the results for both techniques.

*Probabilistic sensitivity analysis*

Beta distributions [10] were used for the parameters of the Bernouilli distributions for participation, sensitivity and specificity. Dirichlet distributions were used for the cancer stage distribution at detection [11].

Participation was sampled from a Bernouilli distribution with the parameter depending on whether the screening round was initial or successive and modeled as a Beta distribution. The parameters of the Beta distributions are shown in Table S1. Modes [12] of these distributions coincided with the point estimation for participation given in the section “Target population”.

Sensitivity and specificity were sampled from Beta distributions at the beginning of each run. Parameters are shown in Table S1. Modes of these distributions coincided with the point estimations obtained from studies and the database of the screening program of Hospital del Mar as described in section ‘Screening events’.

Stage distribution on detection was introduced in the model through Dirichlet distributions [11] by using the absolute numbers of cancers registered in the Cancer Registry at the Hospital del Mar detected through screening or in clinical practice (Table S2).

**References**

1. INEbase. Instituto Nacional de Estadística website. Available: <http://www.ine.es/inebmenu/indice.htm>. Accessed 2012 Sept 24.

2. Perry N, Broeders M, de Wolf C, Tornberg S, Holland R, et al. (2008) European guidelines for quality assurance in breast cancer screening and diagnosis. Fourth edition--summary document. Ann Oncol 19: 614-622.

3. von Karsa L, Anttila A, Ronco G, Ponti A, Malila N, et al. (2008) Cancer screening in the European Union. Report on the implementation of the Council Recommendation on cancer screening – First Report. Luxembourg: Services of the European Commission.

4. Ascunce N, Salas D, Zubizarreta R, Almazan R, Ibanez J, et al. (2010) Cancer screening in Spain. Ann Oncol 21 Suppl 3: iii43-iii51.

5. Rue M, Vilaprinyo E, Lee S, Martinez-Alonso M, Carles MD, et al. (2009) Effectiveness of early detection on breast cancer mortality reduction in Catalonia (Spain). BMC Cancer 9: 326.

6. Lee SJ, Zelen M (1998) Scheduling periodic examinations for the early detection of disease: Applications to breast cancer. Journal of the American Statistical Association 93: 1271-1281.

7. Lee S, Zelen M (2006) A stochastic model for predicting the mortality of breast cancer. J Natl Cancer Inst Monogr 2006: 79-86.

8. Vilaprinyo E, Rue M, Marcos-Gragera R, Martinez-Alonso M (2009) Estimation of age- and stage-specific Catalan breast cancer survival functions using US and Catalan survival data. BMC Cancer 9: 98.

9. Roman R, Comas M, Hoffmeister L, Castells X (2007) Determining the lifetime density function using a continuous approach. J Epidemiol Community Health 61: 923-925.

10. Briggs AH, Claxton K, Sculpher M (2006) Decision Modelling for Health Economic Evaluation. New York: Oxford University Press.

11. Briggs AH, Ades AE, Price MJ (2003) Probabilistic sensitivity analysis for decision trees with multiple branches: use of the Dirichlet distribution in a Bayesian framework. Med Decis Making 23: 341-350.

12. Kerman J (2011) A closed-form approximation for the median of the beta distribution. ArXiv website. Available: <http://arxiv.org/pdf/1111.0433v1.pdf>. Accessed 2011 November 15.
