# Supplementary material for: Budget Impact Analysis of Switching to Digital Mammography in a Population-Based Breast Cancer Screening Program: A Discrete Event Simulation Model
Source: PLoS One. 2014 May 15;9(5):e97459. doi: 10.1371/journal.pone.0097459 (PMC4022526; doi:10.1371/journal.pone.0097459)
Supplement: Table S1 — Beta distributions for probabilistic sensitivity analysis. (DOCX) [file pone.0097459.s002.docx]

**Table S1:** Beta distributions for probabilistic sensitivity analysis.
